# Radial Flow Perfusion Enables Real-Time Profiling of Cellular Metabolism at Low Oxygen Levels with Hyperpolarized ^13^C NMR Spectroscopy

**DOI:** 10.3390/metabo11090576

**Published:** 2021-08-26

**Authors:** Anthony Mancuso, Mehrdad Pourfathi, Ryan M. Kiefer, Michael C. Noji, Sarmad Siddiqui, Enri Profka, Charles N. Weber, Austin Pantel, Stephen J. Kadlecek, Rahim Rizi, Terence P. F. Gade

**Affiliations:** 1 Abramson Cancer Center, University of Pennsylvania, Philadelphia, PA 19104, USA; anmancuso1998@gmail.com; 2 Department of Cancer Biology, University of Pennsylvania, Philadelphia, PA 19104, USA; mnoji@pennmedicine.upenn.edu; 3 Department of Radiology, University of Pennsylvania, Philadelphia, PA 19104, USA; mehrdad@pourfathi.com (M.P.); rkiefer@pennmedicine.upenn.edu (R.M.K.); sarmadsiddiqui@gmail.com (S.S.); eprofka@sas.upenn.edu (E.P.); Charles.weber@uphs.upenn.edu (C.N.W.); Austin.pantel@pennmedicine.upenn.edu (A.P.); stephen.kadlecek@uphs.upenn.edu (S.J.K.); rizi@uphs.upenn.edu (R.R.)

**Keywords:** hyperpolarized ^13^C, DNP, NMR spectroscopy, radial flow, oxygen transport, perfusion, cell immobilization, microcarriers

## Abstract

In this study, we describe new methods for studying cancer cell metabolism with hyperpolarized ^13^C magnetic resonance spectroscopy (HP ^13^C MRS) that will enable quantitative studies at low oxygen concentrations. Cultured hepatocellular carcinoma cells were grown on the surfaces of non-porous microcarriers inside an NMR spectrometer. They were perfused radially from a central distributer in a modified NMR tube (bioreactor). The oxygen level of the perfusate was continuously monitored and controlled externally. Hyperpolarized substrates were injected continuously into the perfusate stream with a newly designed system that prevented oxygen and temperature perturbations in the bioreactor. Computational and experimental results demonstrated that cell mass oxygen profiles with radial flow were much more uniform than with conventional axial flow. Further, the metabolism of HP [1-^13^C]pyruvate was markedly different between the two flow configurations, demonstrating the importance of avoiding large oxygen gradients in cell perfusion experiments.

## 1. Introduction

Hepatocellular carcinoma (HCC) is a growing worldwide problem that lacks effective treatments [1]. Nearly 700,000 new cases are diagnosed each year [2] and the five-year survival rate is less than 15% [3]. The disease is expected to cause one million deaths per year by 2030 [4]. HCC tumors are known to be metabolically heterogeneous. Metabolic energy in HCC is derived from both glycolysis and oxidative phosphorylation (in conjunction with the TCA cycle) [5,6]. The relative importance of these two processes in HCC can vary significantly [5]. Oxygen consumption, which is required for oxidative phosphorylation, produces hypoxic regions that play an important role in metastasis [7,8]. Hypoxia is also known to interfere with the effectiveness of radiation therapy and chemotherapy [9,10]. Imaging-based methods to specifically identify hypoxic regions of tumors currently do not exist. Such methods could significantly improve patient care [11].

**Figure 1:**
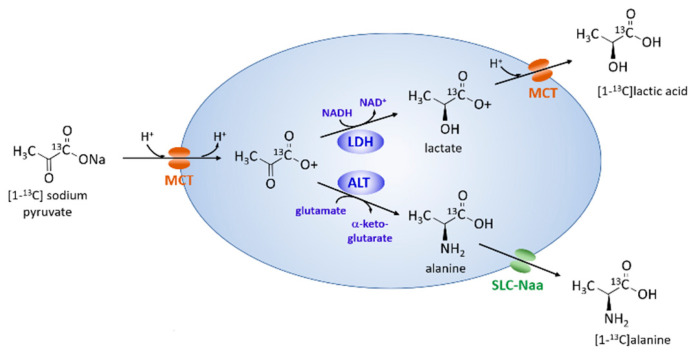
Overview of metabolism of [1-^13^C] pyruvate in cancer cells. MCT = monocarboxylic acid transporters; SLC-Naa = solute carrier protein for neutral amino acids.

Hyperpolarized ^13^C magnetic resonance spectroscopy (HP ^13^C MRS) is a rapidly developing technology with unique capabilities well suited for the determination of metabolic flux in vivo [12,13,14]. It has already been tested in preliminary clinical trials of prostate cancer [15,16] and brain malignancies [17]. HP [1-^13^C] pyruvate has been widely used for studying metabolism because it can detect changes in key enzymes (Figure 1), including lactate dehydrogenase (LDH), alanine aminotransferase (ALT), and pyruvate dehydrogenase (PDH), as well as cytoplasmic levels of NADH [12,13]. It has a long spin lattice relaxation time (T1), which improves its detectability in ^13^C HP imaging [12]. Its value as a specific indicator of hypoxia has not been assessed. Many other hyperpolarized ^13^C compounds have been examined as metabolic tracers [18] but none have been shown to be oxygen-level sensitive. The primary goal of this work was to develop cell culture methods that allow the examination of HP ^13^C substrate metabolism at low controlled oxygen levels.

Several different methods have been used previously for HP cell culture studies. The simplest of these is the rapid injection and mixing of the HP agent with a suspension of cells in a capped NMR tube [19]; however, with this approach, the dissolved oxygen level in the medium cannot be controlled and it declines rapidly after the substrate is added. In addition, anchorage dependent cells cannot proliferate when examined as a suspension, which significantly limits the usefulness of this method. 

A better approach is to inject hyperpolarized substrates into cultures that are immobilized on a stationary matrix. Fixed beds of microcarriers are commonly used for this purpose [20]. The direction of flow through the microcarriers is typically axial, i.e., parallel to the long axis of the NMR tube [21,22,23]; however, this approach has limitations for studies at low oxygen levels. Direct measurement of oxygen concentrations for cancer cells grown in 10-mm [24] or 20-mm [25] tubes have demonstrated that the extent of oxygen depletion in the axial direction is significant. Oxygen levels can drop from 0.20 mM (air saturation) at the inlet to less than 0.1 mM at the outlet when the cell concentration is high [25]. With a lower inlet oxygen level, as would be used to study hypoxia, the percent reduction in oxygen through the cell mass would be even greater. NMR measurements made under such conditions represent the average metabolic state for the entire range of oxygen concentrations and are of limited value. To evaluate ^13^C HP probes as detectors of hypoxia, the range of oxygen concentrations through the cell mass should be kept small. A simple approach to reducing oxygen gradients would be to increase the flow rate of the perfusate; however, increasing the flow rate would also increase the shear stress on the cells and potentially remove them from the microcarriers. 

An alternative approach is to change the direction of flow from axial to radial so that the surface area available to perfuse the cell mass is increased. This scheme also has the advantage that it reduces the time needed to disperse hyperpolarized agents throughout the cell mass, which consequently reduces the loss of ^13^C polarization due to T1 relaxation.

In this study, the feasibility of this method was examined computationally and experimentally for cultured rat HCC cells, which possess a high oxygen consumption rate. The results demonstrate that oxygen gradients can be markedly reduced with radial flow. 

## 2. Results

### 2.1. Calculated Oxygen Profiles for Axial and Radial Flow

**Figure 2:**
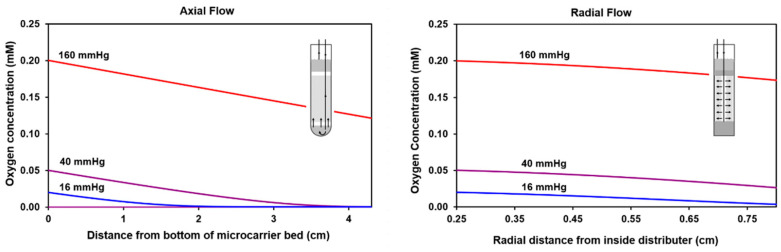
Calculated oxygen profiles for axial and radial flow across the length of the respective cell masses. The flow orientations in the NMR tubes are shown in the small schematic inserts. For both flow configurations, the cell density was assumed to be 1.4 × 10^7^ cells/cm^3^ and the oxygen consumption rate per cell was assumed to be 2.7 × 10^−13^ mmol/s/cell. With axial flow (**left**), the reduction in the oxygen level through the cell mass is much larger than it is with radial flow (**right**). At the lowest inlet level of 0.02 mM (gas phase 16 mmHg of oxygen), the oxygen is essentially depleted less than halfway to the top of the NMR tube with axial flow. In contrast, with radial flow, the oxygen level is more homogeneous and oxygen is available throughout the cell mass.

To determine the potential advantages of using radial flow, oxygen concentration profiles were calculated for both axial and radial flow perfusion schemes at the same cell density. A schematic diagram of the radial flow bioreactor and perfusion apparatus is shown in the methods section below. The computational approach used to determine the oxygen profiles is presented in Appendix A. Profiles were calculated for inlet medium gas phase partial pressures of 160, 40, and 16 mmHg (Figure 2). For the cell culture medium, these partial pressures are associated with dissolved oxygen levels of 0.20, 0.050, and 0.020 mM, respectively. The last two values were chosen because the mean tissue oxygen level in human liver is typically 40 mmHg [26,27], while in hypoxic tumors it is much lower. 

The calculated extent of oxygen depletion with axial flow is significant. At 160 mmHg, the concentration is reduced by 40%. At 40 mmHg, depletion of oxygen is more marked on a percentage basis and near the top of the microcarrier bed, oxygen is fully depleted. At 16 mmHg, the oxygen is completely depleted before the mid-point of the NMR tube. These results demonstrate that sizable oxygen gradients exist with axial flow. With radial flow, the perfusion rate can be increased 3-fold because the surface area for perfusion is 3-fold larger (see Appendix A). The calculated results shown in Figure 2 demonstrate that radial flow significantly reduces the magnitude of oxygen gradients. At both 40 and 16 mmHg of oxygen, radial flow prevents complete depletion of oxygen. These results indicate that radial flow is better suited for low-oxygen HP ^13^C NMR spectroscopy studies than axial flow. 

### 2.2. Experimental Results with Axial Flow

**Figure 3:**
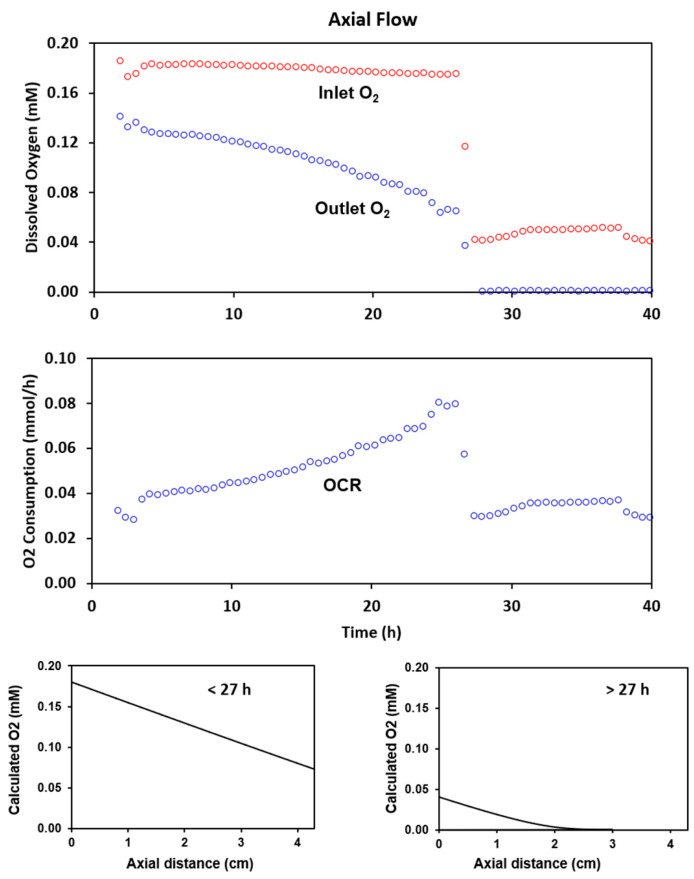
Measured perfusate oxygen levels during growth with axial flow perfusion. Cells were grown on microcarriers for 40 h (**top**). As the cell number increased over the first 27 h, the oxygen consumption rate (OCR) increased to 0.08 mmol/h and the oxygen concentration in the outlet medium declined to approximately 0.07 mM (**middle**). When the inlet oxygen concentration was reduced from approximately 0.18 mM to 0.04 mM, the cell mass became oxygen limited and the oxygen consumption rate dropped to 0.04 mmol/h. Calculated oxygen profiles for inlet oxygen levels of 0.18 mM and 0.04 mM confirm that oxygen is limiting at the lower oxygen concentration (**bottom**).

HR-2 cells were grown with axial perfusion at an inlet dissolved oxygen concentration of approximately 0.18 mM for 27 h. The time courses for the inlet and outlet dissolved oxygen concentrations are shown in Figure 3. Over the 27-h period, the outlet concentration dropped as the oxygen consumption rate of the culture increased to 0.08 mmol/h (middle graph). The calculated oxygen profile in the cell mass indicates that for an inlet oxygen concentration of 0.18 mM (bottom left graph), oxygen should not limit growth.

**Figure 4:**
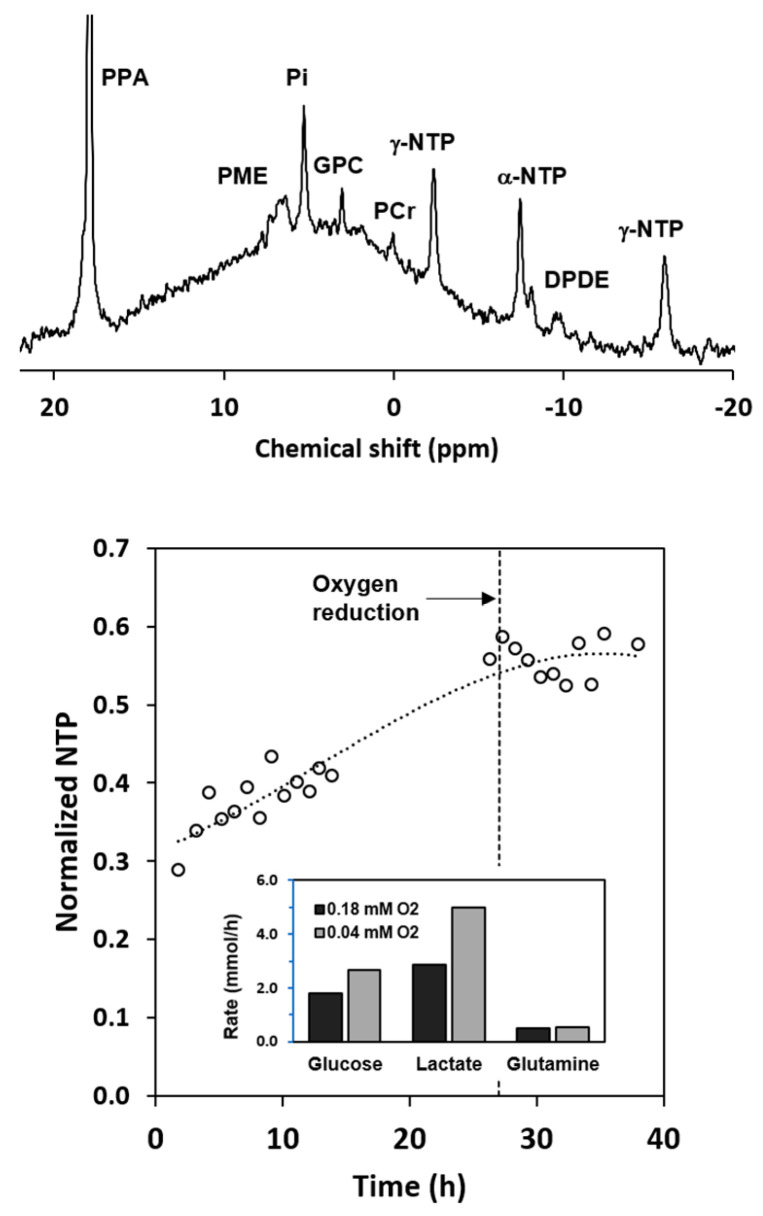
^31^P NMR results for the axial flow experiment summarized in Figure 3. The spectrum at the top of the figure was obtained with 2400 scans at the end of the normoxic period. The time course for the nucleoside triphosphates is shown in the bottom of the figure. Reduction of the inlet medium oxygen level from 0.18 mm to 0.04 mM halted the increase in the NTP level. The glucose and lactate results indicate that reduction of the oxygen level resulted in a metabolic shift toward anaerobic glycolysis.

To evaluate the limitations of axial flow, the inlet oxygen level was reduced to 0.04 mM. The reduction lowered the outlet oxygen concentration to an undetectable level and reduced the oxygen consumption rate to 0.04 mmol/h. The calculated oxygen profile (bottom right) shows that with this change, the cell mass would be very oxygen limited. A typical ^31^P spectrum acquired at the end of the 0.18 mM oxygen period is shown at the top of Figure 4. Prominent resonances include those for phosphomonoesters (PME), inorganic phosphate (Pi), glycerophosphocholine (GPC), phosphocreatine (PCr), the 3 phosphates of nucleoside triphosphates (NTP), and the two phosphates of diphosphodiesters (DPDE). The time course for NTP levels at the two different oxygen concentrations is shown at the bottom of the figure. NTP levels increased markedly over the 27-h high-oxygen period, which is consistent with the oxygen consumption data. When the medium inlet oxygen concentration was reduced to 0.04 mM, the NTP level stopped increasing. Analysis of the extracellular medium, as shown in the inserted histogram, indicated that the rate of glucose consumption and lactate formation both increased under oxygen limitation. The percentage increase in the rate of lactate formation was much larger than the percentage increase in the rate of glucose consumption, indicating that reducing the oxygen concentration resulted in significant anaerobic glycolysis. Little or no change occurred in the rate of glutamine consumption. These results demonstrate the limitations of using axial flow for studying metabolism at low oxygen concentrations.

### 2.3. Metabolism of Hyperpolarized [1-^13^C] Pyruvate with Axial Flow

**Figure 5:**
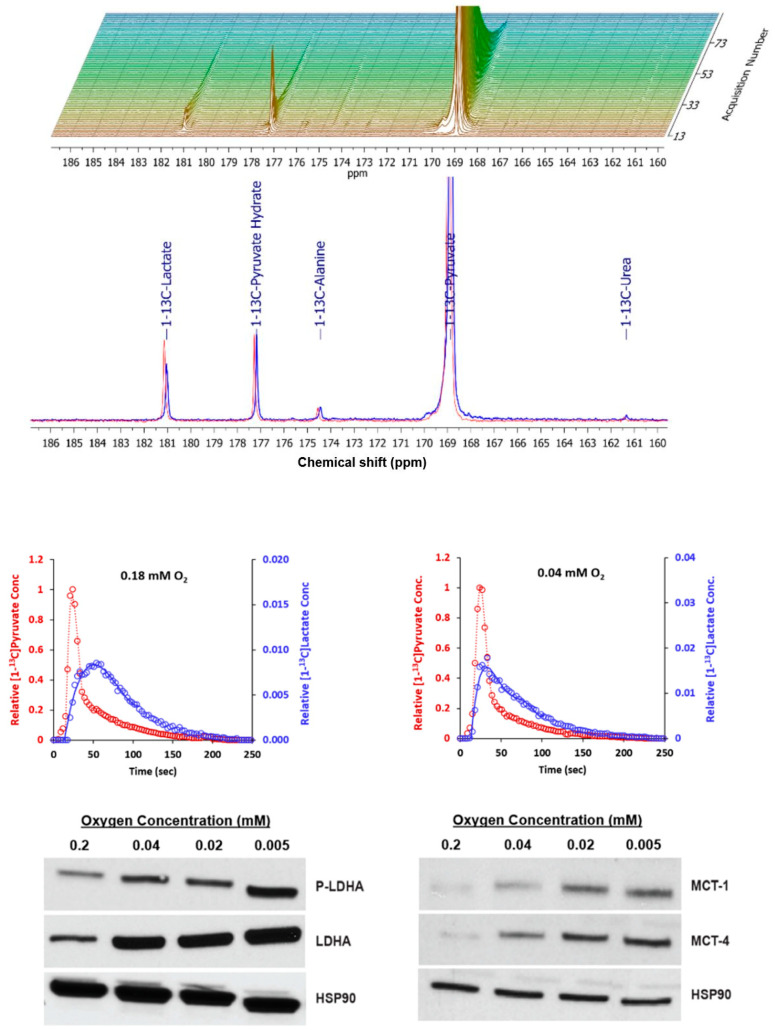
Conversion of hyperpolarized [1-^13^C]pyruvate to [1-^13^C]lactate is impacted by the oxygen level. At 0.18 mM oxygen, [1-^13^C]pyruvate is converted primarily to [1-^13^C]lactate; some is also converted to [1-^13^C]alanine (top stacked plot). The [1-^13^C]urea is a non-metabolic external standard. The blue spectrum below the stacked plot was acquired at the time of the highest level of lactate. Reducing the oxygen level to 0.04 mM produced a higher peak level of lactate (red spectrum). The normalized kinetic profiles for the two injections are shown below the spectra. The smoothed curves are least-square-fit sixth-order polynomials that were used to determine the TTP_LP_. At the bottom of the figure are Western blot results for cells grown at 4 different oxygen levels in 10 cm dishes (bottom). These demonstrate increases in LDHA, phosphorylated LDHA, MCT-1, and MCT-4 at reduced oxygen concentrations.

Two continuous injections with [1-^13^C] pyruvate were conducted during the experiment described in Figure 4. The first was performed while the inlet oxygen concentration was maintained at 0.18 mM; the second was performed at the lower inlet oxygen level. The results are shown in Figure 5. The stacked plot shows the full time course for spectra acquired at 0.18 mM. Shortly after the injection, the [1-^13^C] pyruvate resonance grew rapidly and reached a signal-to-noise ratio in excess of 10,000:1. Both [1-^13^C]lactate and [1-^13^C] alanine were formed. The time to peak intensity for [1-^13^C]lactate was 27 s after the time to peak [1-^13^C]pyruvate (TTP_LP_ = difference between the two peak times). 

At 0.04 mM inlet oxygen, lactate formation was more rapid; the TTP_LP_ was only 8 s. These results are consistent with the increased rate of lactate formation observed by analysis of the extracellular medium (Figure 4). The increased rate of lactate formation could be attributed to an increase in the levels of the enzymes directly involved in the conversion of pyruvate to lactate, specifically lactate dehydrogenase-A (LDHA), its active phosphorylated form (LDHA-P), or the monocarboxylic acid transporters MCT-1 and MCT-4. To examine these possibilities, HR-2 cells were cultured in 10-cm dishes at oxygen levels that would be present in the microcarrier bed with an inlet oxygen level of 0.04 mM. Western analyses for intracellular LDH-A, p-LDHA, MCT-1, and MCT-4 (Figure 5) demonstrated that these enzymes were present at higher levels with reduced oxygen. The increased expression of these enzymes could explain, at least in part, the increase in the rate of formation of lactate.

### 2.4. Radial Flow Improves Oxygen Delivery

**Figure 6:**
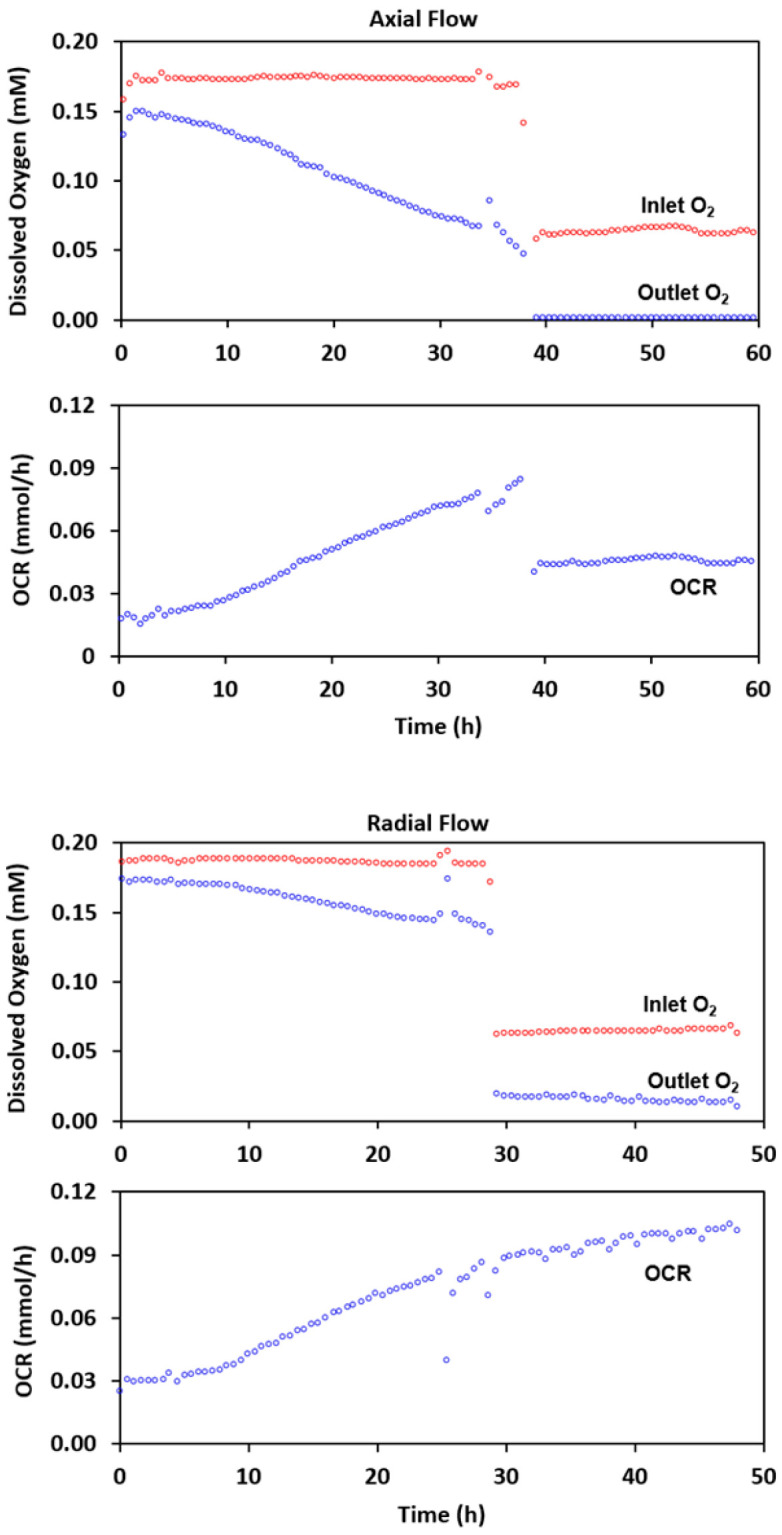
Comparison of axial and radial flow before and after oxygen limitation. Cultures perfused with medium near air saturation were grown to approximately identical levels of oxygen consumption. The difference between the inlet and outlet oxygen levels with axial flow was far greater than for radial flow (top and third graphs); the oxygen consumption rate profiles were similar (second and fourth graphs). When the inlet concentration was reduced to 0.06 mM, the outlet oxygen level with axial flow was below the detectable limit. However, it remained well above that value with radial flow.

Cells were grown on microcarriers in two separate experiments and perfused with either axial or radial flow. The inlet and outlet oxygen measurements for the two experiments are shown in Figure 6.

For the axial flow experiment, cells were grown with an inlet oxygen level of approximately 0.17 mM (top of Figure 6 and Table 1). As the cell density in the NMR tube increased over the course of the 38-h experiment, the outlet oxygen level decreased from 0.15 to 0.05 mM. The oxygen consumption rate reached a maximum of 0.085 mmol/h. Following a reduction to 0.061 mM oxygen in the inlet medium, the outlet concentration dropped to below the detectable limit and the oxygen consumption rate decreased to 0.044 mmol/h. For radial flow, a culture with a similar oxygen consumption rate, 0.086 mmol/h, was subjected to the same change. The outlet oxygen concentration remained well above the detectable limit and the consumption rate did not drop; it was essentially unchanged. With axial flow, the oxygen reduction also caused a marked reduction in NTP level (Table 1). Little change in NTP was observed for radial flow with the oxygen level reduction. 

### 2.5. Radial Flow Improves Viability

**Figure 7:**
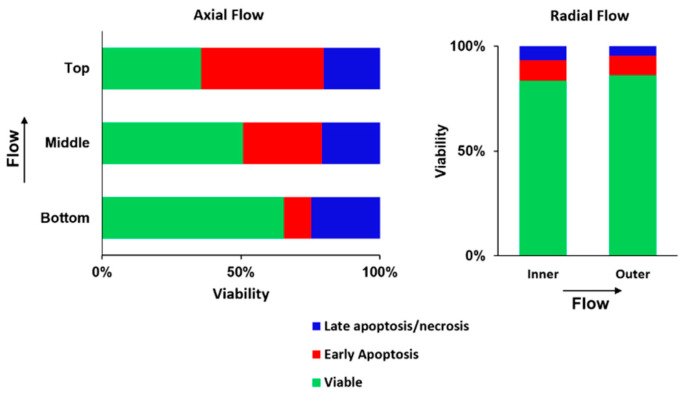
Flow cytometry results for cell perfusion experiments. For the axial flow experiment, 24 h of perfusion at an inlet oxygen concentration of 0.061 mM resulted in significant induction of apoptosis, the extent of which increased with increasing depth in the cell mass (**left**). For radial flow, the extent of apoptosis was small with 0.065 mM inlet oxygen due to improved oxygen transport (**right**).

Induction of apoptosis and necrosis was examined at the end of the experiment described in Figure 7. The cultures were sampled at specific points in the NMR tubes as described in the Methods section. With axial flow, the reduced oxygen level caused marked induction of apoptosis that increased in the direction of flow; that is, in the direction of reduced oxygen. With radial flow, apoptosis was limited throughout the cell mass. The results are consistent with the data shown in Table 1 and further demonstrate that radial flow markedly improves oxygen transport.

### 2.6. Metabolism of Hyperpolarized [1-^13^C] Pyruvate

**Figure 8:**
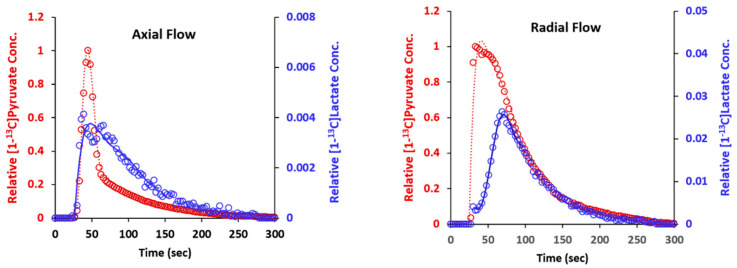
Kinetic profiles of [1-^13^C] pyruvate (red trace) and [1-^13^C] lactate (blue trace) for cells with axial (**left**) and radial (**right**) perfusion in DMEM-I. The smoothed curves are least-square-fit sixth-order polynomials that were used to determine the time-to-peak.

Hyperpolarized [1-^13^C] pyruvate was injected into the culture perfused with radial flow (described in Figure 6) after the inlet oxygen level had been reduced to 0.06 mM. The results are shown on the right in Figure 8. The initial slope for the appearance of [1-^13^C] pyruvate was much higher than it was for axial flow injections, due to the more rapid dispersion of the substrate with radial flow. The TTP_LP_ was 37 s, which was slightly longer than the time observed for the high oxygen level experiment shown in Figure 6. 

For comparison, a culture grown with axial flow that was subjected to oxygen restriction was also examined with hyperpolarized [1-^13^C] pyruvate. The culture had an initial oxygen consumption rate of 0.064 mmol/h. When the oxygen level was lowered to 0.04 mM, the outlet oxygen level dropped to below the detectable limit and the oxygen consumption rate was halved. Under these conditions, the results for metabolism of hyperpolarized [1-^13^C] pyruvate were similar to those shown in Figure 5; that is, the TTP_LP_ was very short (3 s) (Figure 8, left). These results demonstrate that with axial flow, a low inlet oxygen concentration results in metabolism that is consistent with anaerobic glycolysis. In contrast, with radial flow, comparable inlet oxygen levels do not cause a shift to anaerobic glycolysis because the transport of oxygen is greater. 

## 3. Discussion

The results of this work demonstrate that radial flow significantly improves the transport of oxygen in perfused microcarrier beds. This statement is well supported by both the oxygen transfer calculations and the experimental observations. With radial flow, there is a 3-fold increase in the inlet flow cross-sectional surface area, thereby allowing a 3-fold increase in the perfusion rate. The increase in perfusion reduces oxygen gradients in the cell mass and allows NMR spectroscopy measurements to be made under more homogeneous conditions. In contrast, with axial flow, metabolic measurements represent an average over a range of oxygen levels. Such measurements are of lesser value for examining cellular metabolism. In addition, because radial flow allows a higher perfusion rate, both the rate of delivery of the hyperpolarized substrate to the NMR tube and its distribution in the microcarrier bed are enhanced. Both factors allow for more efficient use of the initial polarization. 

Further gains in oxygen concentration homogeneity would be possible with a larger inner diameter of the microcarrier bed. For example, if the inner and outer diameter of the microcarrier bed were increased to 9 mm and 20 mm, respectively, the perfusate rate could be nearly doubled over the radial flow rate used in this study. Such a change would reduce the size of the oxygen gradients by nearly half. The larger microcarrier bed would require the use of a 25-mm NMR tube with a similarly sized liquids probe. The loss in sensitivity due to the larger NMR coil diameter (a linear function) would be more than offset by the increase in cell number (a quadratic function). The central distributer could be composed of polyetherimide (Ultem^TM^) rather than polyethylene to reduce susceptibility contrast in the center of the bioreactor. Liquid probes for 25-mm NMR tubes are readily available from commercial manufacturers for spectrometers with 89-mm clear bores. 

The cell line used for this study, HR-2, has an extremely high oxygen consumption rate, which is common for HCC cell lines [28,29]. Non-hepatic cancer cell lines that have been examined with cell perfusion NMR, including glioblastoma [30] and melanoma [31]), have oxygen consumption rates that are one-fifth to one-tenth that for HR-2 cells. With such cells, much more homogeneous oxygen profiles could be maintained. Radial flow could be greatly beneficial for studying such cell types under hypoxia. 

Other means for perfusing cells inside NMR spectrometers have been examined with conventional NMR [20]. Any approach with axial flow in an NMR tube, such as porous microcarriers or entrapment in hydrogels, would have the same problems with axial gradients as described in this manuscript, with additional gradients due to diffusional limitations inside the immobilizing agent. For conventional hollow fiber bioreactors, axial gradients can be markedly reduced by using high luminal flow rates, since the cell mass is protected from shear stress by the fiber walls [32]; however, with hollow fibers, transport of the hyperpolarized substrate into the cell mass would be slowed by the fiber wall. If the cell density in the extraluminal space were high, transport across the wall would be predominantly diffusive [33]; that is, there would be little or no Starling flow. Given that the diffusivity of pyruvate in water is ~1 × 10^−5^ cm^2^/s [34], the diffusivity of pyruvate in a typical hollow fiber membrane (D_p_) would be approximately 3 × 10^−6^ cm^2^/s [20]. For fibers with a wall thickness, w, of 100 µm, the characteristic time for pyruvate diffusion [35], τ = w/2D_p_, would be 17 s; thus, diffusion of the hyperpolarized agent across the fiber membrane would be associated with a significant loss of polarization. Accordingly, hollow fiber bioreactors may not be well suited for work with hyperpolarized NMR.

An alternative to changing the perfusion geometry would be to change the way the NMR data are acquired. For example, with axial flow, HP ^13^C metabolic data could be spatially encoded in the axial direction with either frequency encoding or slice selection. With this approach, the range of oxygen concentrations for the acquired ^13^C data could be greatly reduced. Such an approach would compromise the signal to noise but would be simple to implement. It would also allow the simultaneous acquisition of ^13^C NMR spectra at multiple oxygen concentrations. 

The number of previously published cell perfusion NMR studies with low oxygen concentrations is very limited [32,36,37]; however, in one recent study, the effects of low oxygen levels on the conversion of hyperpolarized [1-^13^C]pyruvate to [1-^13^C]lactate were examined quantitatively with and without inhibition of MCT-1 [38]. The kinetics of the conversion were analyzed with a high degree of analytical rigor. The authors reported that the conversion of pyruvate to lactate was not limited by LDH but rather by MCT-1 transport. They also observed that hypoxia increased the rate of conversion of pyruvate to lactate, which is consistent with the findings of the current study; however, unlike the current study, the authors injected the HP-^13^C pyruvate as a bolus without removing the oxygen before it reached the cell mass. Further, no oxygen-level monitoring or control was used, so from an oxygen point of view, the results can only be interpreted semi-quantitatively. Nevertheless, this study demonstrates the potential of HP-^13^C NMR to detect metabolic changes associated with hypoxia.

## 4. Materials and Methods

### 4.1. Cell Culture

All studies were conducted with rat HCC cells (HR-2) obtained from Dr. Istvàn Blaszsek of Hôpital Paul-Brousse, Villejuif, France [39]. Cells were routinely maintained in Dulbecco’s modified Eagle’s medium (DMEM) containing 17 mM glucose, 4 mM glutamine, and 20 mM HEPES. The medium was supplemented with 10% fetal bovine serum (FBS, Gemini, West Sacramento, CA, USA) and 1% penicillin–streptomycin (DMEM-S). DMEM-S was also used for cell perfusion unless otherwise stated. Ischemic medium (DMEM-I) was the same as DMEM-S, except it contained 1% FBS, 1 mM glucose, and 0.5 mM glutamine. Cultures were maintained in 10-cm tissue-culture-treated (TCT) dishes prior to use in experiments. For inoculation of microcarriers, cells were grown in 15-cm TCT dishes to less than 100% confluency. 

### 4.2. Microcarrier Preparation

Collagen-coated non-porous polystyrene microcarriers were used for all studies (Sigma-Aldrich, St. Louis, MO, USA). The microcarriers were prepared as described by the vendor (Sigma). Cells were grown on the microcarriers inside flat-bottom (120 cm^2^) 1-L borosilicate glass bottles (Corning, Corning, NY, USA), which were pre-coated with Sigmacoat (Sigma-Aldrich). A total of 10^7^ cells were used to inoculate each 1-L bottle containing 1.5 gm of microcarriers. Cells were grown on the surfaces of the microcarriers for 18 h. Subsequently, a combined total of 9 gm of microcarriers was transferred to a 2-L spinner flask that contained 1 L of DMEM-S. The microcarriers were used for NMR experiments when approximately 50% of their surfaces were covered with cells.

### 4.3. Cell Perfusion

**Figure 9:**
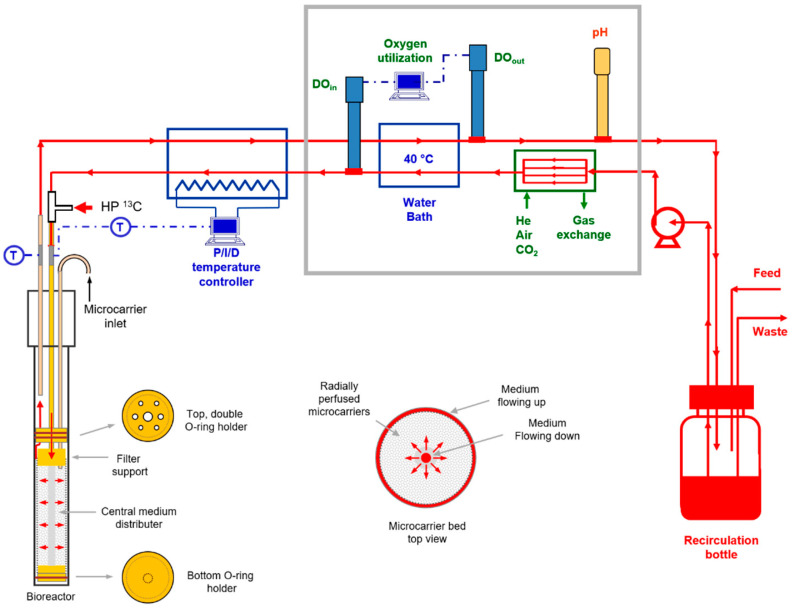
Radial flow perfusion system for microcarrier-immobilized cells. The perfusion system was similar to the one described previously for axial flow [25]. The medium was circulated through a continuous loop that included a peristaltic pump, a gas exchange module, two oxygen probes (one for the inlet medium and one for the outlet), a P/I/D-controlled heater, the NMR tube with microcarrier-immobilized cells, a secondary temperature probe, a computer for continuous calculation of the oxygen consumption rate, a pH probe, and a medium recirculation bottle. To prevent depletion of metabolites in the recirculating medium, a continuous feed of fresh medium was used in conjunction with continuous waste removal.

Microcarriers were perfused inside sealed NMR tubes with temperature, oxygen, and pH control as described previously [25], with some modifications (Figure 9). The medium flowed through a continuous loop and was re-oxygenated through four parallel 5-m-long sections of silicone tubing (2.06 mm O.D. × 1.02 mm I.D.) inside a sealed glass cylinder. The relative amounts of air, helium, and CO_2_ flowing through the glass cylinder were adjusted with rotameters to control the dissolved oxygen level and the pH of the medium. Helium was used rather than nitrogen to avoid degassing of the medium and consequent bubble formation. Inline polarographic oxygen probes (Mettler-Toledo, Columbus, OH, USA) were used at the inlet and outlet of the NMR tube. An inline pH probe (Mettler-Toledo) was used to continuously monitor the pH of the medium. All three probes were housed in well-sealed polysulfone casings that were constructed at our institution. 

The medium was continuously recirculated into a 100 cm^3^ glass bottle inside a small incubator (located 4 m from the magnet bore). The incubator also contained the oxygen and pH probes, which were found to be sensitive to temperature variations in the lab. The temperatures at both the inlet and outlet of the NMR tube were monitored with copper–constantan thermocouples that did not directly contact the sterile medium. The NMR temperature was controlled with a proportional–integral–derivative controller (Omega Engineering, Norwalk, CT, USA) at 36.5 +/− 0.5 C. The controller adjusted the current in a 4-m-long electrical resistance heater that traced the perfusion line between the incubator and the NMR magnet. The temperature, pH, and oxygen measurements were continuously acquired with analog-to-digital converters (USB-2008, National Instruments, Austin, TX, USA) interfaced to a computer running Labview 7.5 (National Instruments). A high-precision multi-channel Rainin Dynamax pump (Mettler-Toledo Rainin, Oakland, CA, USA) was used to supply fresh medium to, and remove spent medium from, the recirculation bottle at a rate of 0.014 cm^3^/s. Nearly all tubing in the perfusion system between the oxygen probes and the NMR tube was polyether ether ketone (PEEK, 3.2 mm O.D. × 2.4 mm I.D.), which has an extremely low oxygen permeability [40]. The only non-PEEK tubes were very short sections of flexible, thick-walled, Pharmed^®^ tubing (2.0 mm I.D., Cole Parmer, Vernon Hills, IL, USA) that were used to make air-tight connections. The entire perfusion system was sterilized in a steam autoclave prior to each experiment.

### 4.4. NMR Spectroscopy

NMR spectra were acquired with an Oxford 9.4 Tesla 8.9-cm-bore magnet (Oxford Instruments, Abingdon, UK) interfaced to a 400 MHz Varian DirectDrive Console (Palo Alto, CA, USA). A 20-mm broadband liquids probe (Varian) was used to acquire ^31^P and ^13^C spectra at 162.1 and 100.7 MHz, respectively. ^31^P spectra were acquired with 60° pulses, a repetition time of 1 s, 4096 points, and a spectral width of 15,000 Hz. ^13^C spectra were acquired with a single 12° pulse, a repetition time of 3 s, 8192 points, and a spectral width of 25,000 Hz. WALTZ-16 decoupling was used during signal acquisition to eliminate ^1^H couplings. 

### 4.5. NMR Flow Cell Designs

For axial flow perfusion, the microcarriers were held between two porous high-density polyethylene (HDPE) filters in a standard screwcap 20-mm NMR tube (Wilmad, Vineland, NJ, USA) [25]. Both were sealed against the NMR tubes with 15 mm I.D. by 1.6-mm-thick silicone o-rings. For radial flow, the microcarriers were held between two cylindrical HDPE filters (inner filter: 1.6 mm I.D. × 5 mm O.D.; outer filter: 16 mm I.D. × 17.4 mm O.D.). The NMR tube had an inner diameter of 18.4 mm and an outer diameter of 20.0 mm. The filters were held in place with custom-manufactured polyetherimide caps. Both had an outer diameter of 16 mm. The upper polyetherimide cap hung from a polyetherimide disc with a 17.5 mm O.D. The two polyetherimide pieces were joined (with glue) by a polyetherimide tube measuring 25 mm long, with a 1.6 mm I.D. and a 4.0 mm O.D. Medium flowed down through the inner polyetherimide tube, the inner HDPE cylinder, radially across the cell mass, and out of the NMR tube through the annular space between the outer cylinder and the NMR tube. It flowed past the upper polyetherimide disc through four 1.6-mm-diameter holes. Two additional holes measuring 2.0 mm in diameter were used for PEEK tubing. One was used to introduce the microcarriers into the NMR tube. The other was used to hold an external standard in the NMR-detectable volume. 

### 4.6. Hyperpolarization and Deoxygenation of [1-^13^C] Pyruvic Acid

Metabolic studies were conducted with [1-^13^C]pyruvic acid (Cambridge Isotopes, Tewksbury, MA, USA) that was polarized with a HyperSense DNP Polarizer (Oxford Instruments). Typically, 112 mg of pyruvic acid (99% ^13^C enriched) was mixed with OX63 radical at a 160:1 molar ratio. Polarization at 94.07 GHz required approximately 1 h. After polarization, the frozen pyruvic acid glass was solubilized and purged from the HyperSense sample with 6.0 cm^3^ of aqueous buffer that contained 85 mM sodium hydroxide, 40 mM 4-(2-hydroxyethyl)-1-piperazineethanesulfonic acid, and 0.42 mM EDTA (melt buffer).

**Figure 10:**
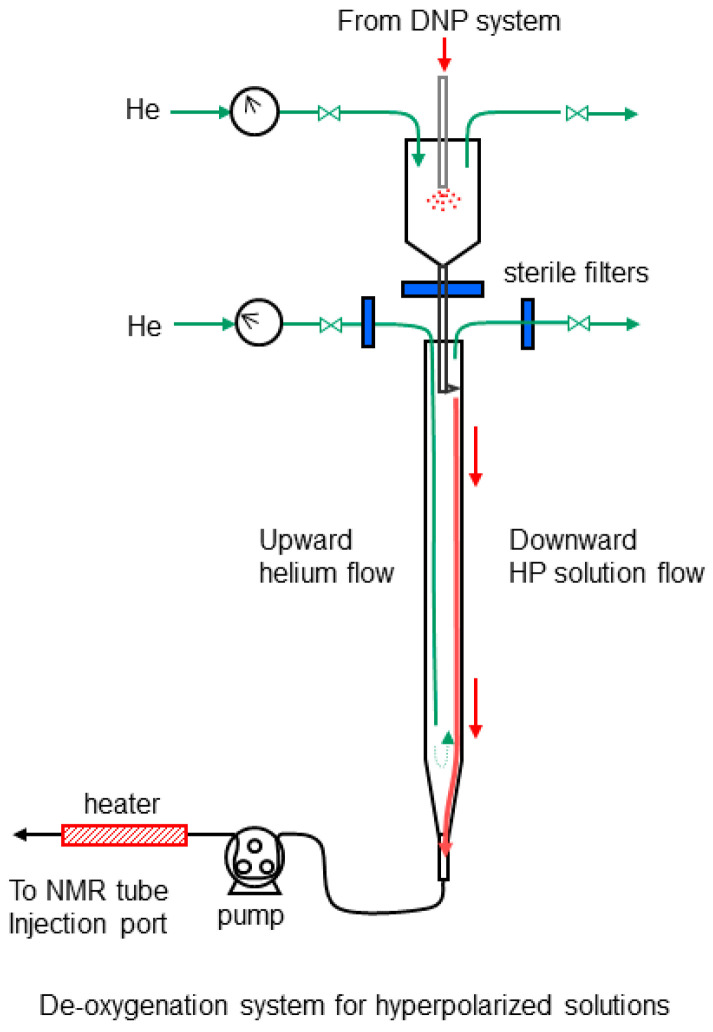
System used for injecting oxygen depleted solutions containing hyperpolarized substrates into the NMR tube. Two stages of helium sparging were used to ensure thorough oxygen removal. Sterile filters were used for both the helium and the hyperpolarized solutions before the second stage.

Hyperpolarized substrates were injected as continuous streams rather than as boluses, since bolus injections perturb both the oxygen level and the temperature of the cell mass. After flowing out of the HyperSense system, the sodium pyruvate solution was de-oxygenated in a device constructed in our laboratory (Figure 10). In the first stage, the sodium pyruvate solution flowed into a vented 50-cm^3^ glass cylinder that was filled with gaseous helium and 2 cm^3^ of the aqueous buffer. The final concentration of [1-^13^C]sodium pyruvate was 85 mM. After the pressure from the HyperSense system was fully vented, helium was used to force the partially de-oxygenated solution through a sterile filter. The solution subsequently flowed down the side of an 18-cm-long glass column (15 mm I.D.), where counter-current helium flow was used to remove additional oxygen. The de-oxygenated solution was injected into the continuously flowing perfusate with a peristaltic pump (Cole-Parmer) at the inlet of the NMR tube (Figure 9). The injection line comprised thick-walled PEEK tubing with an inner diameter of 0.76 mm (Small Parts, Inc, Logansport, IN, USA). The peristaltic tubing was FDA-compliant Viton tubing (Cole-Parmer, 0.8 mm I.D.), which has extremely low oxygen permeability. The HP injection flow rate was 10% of the total perfusate rate for all experiments. The hold-up volume of the injection line was 1.5 cm^3^. The line was filled with sterile cell culture medium from the perfusion line before each injection. The medium was pre-warmed as it was injected with an electrical resistance heater that was wrapped around the injection line. To inject the hyperpolarized substrate, the main perfusion pump was switched off and the HP injection pump was simultaneously turned on at a rate of 0.2 cm^3^/s (identical to the perfusion rate). This step was used to purge the dead volume of the injection line. After 12 s, the HP injection rate was reduced to 0.02 cm^3^/s and the perfusion pump was immediately turned on at a rate of 0.18 cm^3^/s. The two solutions were mixed in a Y-fitting at the inlet to the NMR tube. This procedure did not perturb the inlet medium temperature. 

### 4.7. Small Metabolite Analysis

Glucose, lactate, and glutamine concentrations were determined with an immobilized enzyme analyzer (YSI 2950, YSI Inc., Yellow Springs, OH, USA). Metabolic fluxes were estimated as described previously [41].

### 4.8. Flow Cytometry

Following the completion of each perfusion experiment, a small sample of microcarriers was removed from the microcarrier bed with a pipette. The microcarriers were centrifuged at 2000 rpm for 4 min. The supernatant was aspirated and the cells were dissociated from the microcarriers with 0.25% trypsin. The trypsin was quenched with DMEM-S and the microcarriers were removed from the cell suspension with a 40-μm cell strainer (Fisher Scientific, Hampton, NH, USA). The cell suspension was centrifuged at 2000 rpm. The resultant cell pellet was re-suspended in 100 μL of diluted binding buffer and stained with Annexin V-FITC and propidium iodide (PI) (BD Biosciences, San Jose, CA, USA). The cell suspension was analyzed with an Accuri Flow Cytometer (Becton Dickinson, Franklin Lakes, NJ, USA) until a total of 125,000 counts were accumulated. Cellular viability was determined using FlowJo software (FlowJo LLC, Ashland, OR, USA). Cells that stained positive for Annexin V-FITC and negative for PI were counted as apoptotic.

### 4.9. Western Analysis

HR-2 cells were grown in DMEM-S in 10-cm dishes for 24 h with 0.20 mM oxygen before they were moved to lower oxygen concentrations (0.04, 0.02, and 0.005 mM) for 48 h before harvest. At the time of harvest, cells were scraped into radioimmunoprecipitation lysis buffer (Thermo Fisher Scientific, Waltham, MA, USA) and sonicated. Protein levels in the lysates were quantified by BCA (Thermo Fisher Scientific). Western blot analysis was conducted with an electrophoresis chamber and semi-dry turbo transfer system (BioRad, Hercules, CA, USA). The antibodies used were: LDHA: mAB #3582, P-LDHA pAB #8176, HSP90 (mAB #4877) (Cell Signaling Technology, Danvers, MA, USA); MCT-1 (pAB: T-19, sc-14917) and MCT-4 (pAB: H-90, sc-50329) (Santa Cruz Biotechnology, Dallas, TX, USA).

### 4.10. Spectral Analyses

Spectral data were quantified by numerical integration with MNova (Mestrelab Research, Santiago de Compostela, Spain). The time to peak concentration of [1-^13^C]lactate TTP_LP_ was determined by fitting the time course of the [1-^13^C]pyruvate and [1-^13^C]lactate data to sixth-order polynomials. 

## 5. Conclusions

The methods presented in this work will be useful to identify new HP ^13^C substrates that are sensitive identifiers of hypoxia in solid cancers. They allow perfused cells to be cultured at reduced oxygen levels under controlled conditions. They are an improvement over previous methods in that oxygen levels in the cell mass are more homogeneous and the injection of the hyperpolarized material is accomplished without significant perturbation of the perfusate oxygen level or temperature.

## Figures and Tables

**Table 1:** Effects of oxygen limitation on oxygen consumption rate and total NTP levels for axial and radial flow. Cells were perfused with DMEM-S before the oxygen reduction and DMEM-I after the oxygen reduction.

Axial			Radial		
	Initial	Post O_2_ Reduction		Initial	Post O_2_ Reduction
Inlet O_2_ (mM)	0.168 ± 0.012	0.061 ± 0.001	Inlet O_2_ (mM)	0.183 ± 0.001	0.065 ± 0.003
Outlet O_2_ (mM)	0.051 ± 0.000	<0.0002	Outlet O_2_ (mM)	0.139 ± 0.000	0.014 ± 0.000
OCR (mmol/h)	0.083 ± 0.000	0.044 ± 0.001	OCR (mmol/h)	0.086 ± 0.003	0.097 ± 0.001
NTP (normalized)	0.14 ± 0.01	0.05 ± 0.01	NTP (normalized)	0.09 ± 0.01	0.08 ± 0.01

## Data Availability

Data acquired for this study will be made available upon reasonable request to the corresponding author.

## Appendix A

To determine the potential advantages of using radial flow, oxygen concentration profiles were calculated for both axial and radial flow perfusion schemes at the same cell density. A critical parameter for the calculations is the medium perfusion rate, expressed here as the superficial velocity (volumetric flow rate/void area). Experimentally, we observed that with a superficial velocity of 0.2 cm/s in 20-mm NMR tubes, HCC cells grow well in the microcarrier bed; shear stresses do not remove cells from the microcarriers or inhibit their growth. For comparison, in a previous study with perfused cells, 0.13 cm/s was commonly used with 10-mm NMR tubes [20].

The Reynolds number for flow in the microcarrier bed can be calculated from:

(A1) $$Re=\frac{\rho V_{s}d_{m}}{\varepsilon \mu }$$

where *ρ* is density of the medium at 37 °C (0.99 gm/cm^3^), *V_s_* is the superficial velocity of the medium in microcarrier bed, *d_m_* is the mean microcarrier diameter (170 microns), *μ* is the viscosity of the medium at 37 °C (0.70 cp), and *ε* is the void fraction in the microcarrier bed (0.40). Substitution of these values results in a Reynolds number of 0.47, indicating that the flow is laminar. Reynolds numbers of less than 1.0 are also commonly used for perfusing hepatocytes in bioartificial livers [42].

In order to estimate the oxygen concentration profiles in the calculations below, a superficial velocity of 0.2 cm/s was used. The axial dispersion of oxygen due to parabolic flow was neglected, which is appropriate for low Reynolds number flow in fixed beds [43].

To estimate the oxygen concentration profile in the microcarrier bed, the equation of continuity for a binary mixture was used [44]:

(A2) $$v\cdot \nabla \left[O_{2}\right]=D_{O_{2}}\nabla^{2}\left[O_{2}\right]+R_{O_{2}}$$

In this equation, v is the medium velocity vector, [O_2_] is the concentration of oxygen, *D*_O_2__ is the diffusivity of oxygen in cell culture medium, and $R_{O_{2}}$ is the consumption rate of oxygen. If the extent of growth on the microcarriers is limited to a monolayer, no significant diffusion limitation in the cell mass or the boundary layer of the perfusate flowing past the cells will exist. For the reaction term, the oxygen consumption rate can be assumed to follow Michaelis–Menten kinetics [45]. With these assumptions, and the use of cylindrical coordinates, Equation (A2) becomes:

(A3) $$v_{r}\frac{\partial \left[O_{2}\right]}{\partial r}+v_{\theta }\frac{1}{r}\frac{\partial \left[O_{2}\right]}{\partial \theta }+v_{z}\frac{\partial \left[O_{2}\right]}{\partial z}=\frac{-Xq_{O_{2,max}}\left[O_{2}\right]}{\left(k_{m}+\left[O_{2}\right]\right)}$$

where *X* is the average cell density in the NMR tube *q*_O2, *max*_ is the maximum oxygen consumption rate per cell, and *k_m_* is the Michaelis constant for oxygen consumption. The first two terms can be neglected because there is no net convective flux in the r or θ directions. The remaining two terms for Equation (A3) can be separated and integrated:

(A4) $$\int \frac{v_{z}\left(k_{m}+\left[O_{2}\right]\right)}{Xq_{O_{2,max}}\left[O_{2}\right]}d\left[O_{2}\right]=-\int dz$$

The medium velocity in the *z* direction can be expressed in terms of the medium feed rate (*F*), the cross-sectional area of the NMR tube, and the void fraction of the microcarrier bed: *v_z_* = *F*/(επR^2^). Substituting this expression, integrating and applying the initial condition at *z* = 0, [O_2_] = [O_2_]_o_ results in:

(A5) $$z=\frac{F({\left[O_{2}\right]}_{o}-\left[O_{2}\right]+k_{m}\ln\left({\left[O_{2}\right]}_{o}/\left[O_{2}\right]\right))}{\varepsilon \pi R^{2}Xq_{O_{2,max}}}$$

Although, this result is not an explicit function of the oxygen concentration, it allows the calculation of the axial oxygen concentration profile in the z direction. For experiments in our laboratory, standard parameters were: *F* = 0.2 cm^3^/s and *R* = 0.9 cm. Additionally, for HCC cells, we commonly observed *X* = 1.4 × 10^7^ cells/cm^3^ and *q*_O2, *max*_ = 2.7 × 10^−13^ mmol/s/cell. The *k_m_* value for HCC cells was not readily available in the published literature; a *k_m_* of 5 mmHg (0.00624 mM) has been reported for perfused hepatocytes [42] and was assumed for the results shown in Figure 2.

For radial flow, Equation (A3) simplifies into:

(A6) $$v_{r}\frac{d\left[O_{2}\right]}{\mathrm{dr}}=R_{O_{2}}$$

Given that the medium is distributed through the cell mass from a central distributer, *v_r_* = *F*/(ε2πrh), where h is the height of the central distributer. Substituting this expression and the Michalis expression for *R*_O2_ into Equation (A6) gives:

(A7) $$\left(\frac{F}{\varepsilon 2\pi \mathrm{rh}}\right)\frac{d\left[O_{2}\right]}{\mathrm{dr}}=\frac{-Xq_{O_{2,max}}\left[O_{2}\right]}{k_{m}+\left[O_{2}\right]}$$

Separation of the variables, integration and application of the initial condition (at $r=r_{i}$, [O_2_] = [O_2_]_o_) gives the following result:

(A8) $$r={\left(r_{i}{}^{2}+\frac{F({\left[O_{2}\right]}_{o}-\left[O_{2}\right]+k_{m}\ln\left({\left[O_{2}\right]}_{o}/\left[O_{2}\right]\right))}{\varepsilon \pi hXq_{O_{2,max}}}\right)}^{\frac{1}{2}}$$

With radial flow, the surface area available to perfuse the microcarrier bed is increased approximately 3-fold relative to axial flow; hence, for the same shear stress at the microcarrier surface, the perfusion rate, F, can be increased 3-fold to 0.6 cm^3^/s. The radial oxygen concentrations were determined with h = 4.3 cm and $r_{i}=$ 0.25 cm for the same three inlet oxygen concentrations as for axial flow. The results are shown at the bottom of Figure 2.
